# Reviewing 15 years of experience with sirolimus

**DOI:** 10.1186/s13737-015-0028-6

**Published:** 2015-12-22

**Authors:** Helio Tedesco Silva, Claudia Rosso Felipe, Jose Osmar Medina Pestana

**Affiliations:** Nephrology Division, Hospital do Rim, Escola Paulista de Medicina, Universidade Federal de São Paulo, São Paulo, Brazil

## Abstract

Here, we review 15 years of clinical use of sirolimus in our transplant center, in context with the developing immunosuppressive strategies use worldwide. The majority of studies were conducted in de novo kidney transplant recipients, using sirolimus (SRL) in combination with calcineurin inhibitors (CNIs). We also explored steroid (ST) or CNI-sparing therapies, including CNI minimization, elimination, or conversion strategies in combination with mycophenolate (MMF/MPS). Pooled long-term outcomes were comparable with those obtained with CNI and antimetabolite combination. Surprisingly, there are still several areas that need further investigation to improve the risk/benefit profile of SRL in kidney transplantation, including pharmacokinetic/pharmacodynamic drug-to-drug interaction with cyclosporine (CsA) or tacrolimus (TAC), mechanisms of SRL-associated adverse reactions and combinations with other drugs such as belatacept and once-daily TAC, possibly leading to improved long-term adherence. These studies, along with others investigating the benefits of SRL associated lower viral infections and malignancies, are essential as we do not expect the introduction of new immunosuppressive drugs in the near future.

## Introduction

Beginning on June 1999, we started exploring alternative immunosuppressive regimens using sirolimus (SRL). We began our experience by combining SRL with progressive reduction in cyclosporine (CsA) exposure in de novo kidney transplant recipients. This initial experience was followed by studies exploring SRL exposures combined with reduced CsA exposure in black patients, early CsA minimization or elimination strategies and the use of SRL in calcineurin inhibitor (CNI)-free regimens combined with mycophenolate (MMF). With the increasing use of tacrolimus (TAC) in de novo kidney transplant recipients, we also conducted a head-to-head comparison of SRL with MMF followed by another study comparing steroid (ST) or TAC withdrawal in kidney transplant recipients receiving de novo therapy with SRL. Later, we began to explore conversion strategies, either late or early conversions from CNI to SRL.

## De novo kidney transplant recipients

In our first open-label randomized trial, we compared the safety and efficacy of 2mg fixed daily doses of SRL with 2 mg/kg fixed daily doses of azathioprine (AZA) in living related renal allograft recipients receiving CsA and ST [1]. Because first reports suggested the potentiation of CsA nephrotoxicity by SRL [2, 3], we attempted to implement a small reduction in CsA exposure. In this study, CsA concentrations were lower in patients receiving SRL compared to AZA from week 4 (247 vs. 309 ng/mL, *p* = 0.04) to month 12 (143 vs. 188 ng/mL, *p* = 0.045). The incidence of the primary composite endpoint (biopsy confirmed acute rejection, graft loss, or death) was lower in SRL group at 3 months (0 vs. 17.1 %, *p* = 0.025) but not at 12 months (11.4 vs. 14.3 %, ns). The relatively small reduction in CsA exposure was associated with no difference in mean serum creatinine at 12 months (1.8 ± 0.6 vs. 1.6 ± 0.6 mg/dL, *p* = 0.23) but the small sample size may not had sufficient power to detect the small difference observed. Therefore, we decided to expand our experience to reach 90 patients receiving 2mg fixed daily doses of SRL [4]. At 12 months, mean whole blood CsA trough concentrations were 100 ng/mL in patients receiving SRL and 200 ng/mL in patients receiving AZA. Retrospective analysis showed that mean whole blood SRL trough concentrations increased from day 7 to months 1 and 12 (5.2 ± 3.1 vs. 7.5 ± 3.6 vs. 8.4 ± 6.0 ng/mL, *p* <0.0001) with a tenfold interindividual variability, ranging from 2.5 to 23.5 ng/mL. There was no difference in 1-year composite efficacy endpoint comparing SRL and AZA groups (18 vs. 20 %) or in the incidence of biopsy-proven acute rejection (14.4 and 14.3 %). Importantly, even with higher sample size, we were unable to detect difference in mean serum creatinine (1.65 ± 0.46 vs. 1.60 ± 0.43 mg/dL, *p* = 0.48) or in mean calculated creatinine clearances (61 ± 15 vs. 62 ± 13 mL/min, *p* = 0.58) at 1 year. At that time, we concluded that the use of SRL and reduced CsA exposure was effective in preventing acute rejection and preserving allograft function.

Brazil has a highly miscegenated population with African ancestry that is at an increased risk for renal allograft failure [5]. Knowing that studies had demonstrated that black patients requires higher doses of SRL to achieve comparable efficacy compared to the Caucasian population [6], we designed a study to identify optimal therapeutic SRL concentrations in black kidney transplant recipients receiving reduced CsA exposure and prednisone [7]. Black patients received CsA, ST, and 5mg fixed doses of SRL till day 7 when they were randomized to maintain whole blood SRL trough concentrations between 8 and 12 or 15 and 20 ng/mL. There was no difference in mean whole blood CsA trough concentrations at months 1 (182 ± 86 vs. 162 ± 87 ng/mL) and 12 months (62 ± 43 vs. 59 ± 52 ng/mL). At 6 months, mean whole blood SRL trough concentrations were 10.8 ± 5.8 vs. 18.0 ± 6.1 ng/mL (*p* <0.001). The incidence of biopsy-proven acute rejection was higher in the lower SRL concentration group (18 vs. 8 %). Mean calculated creatinine clearance was higher in the lower SRL concentration group (64.5 ± 17 vs. 54.4 ± 14.7 mL/min, *p* = 0.011) despite higher incidence of acute rejection. The incidence of post-transplant diabetes mellitus was 13 % and no CMV disease was observed. Higher SRL concentrations were associated with higher efficacy but lower renal function, further emphasizing the complex interaction between these two drugs [8].

To further investigate the interaction between SRL and CsA, we performed a sequencial pharmacokinetic study at days 7, 30, and 90 in kidney transplant recipients receiving 2- or 5-mg fixed daily doses of SRL. Both SRL and CSA showed moderate to high inter (39 to 70 and 33 to 52 %) and intra (30 to 41 and 25 to 43 %) subject variability, respectively. A threefold increase in SRL concentrations (4.7 to 14.3 ng/mL) resulted in 37 % increase in mean CSA AUC (6,654 to 9,133 ng.h/mL, *p* = 0.007). Similarly, a 1.8-fold increase in CSA concentrations (190 to 345.5 ng/mL) resulted in 115 % increase in mean SRL AUC (199.8  to 428.9 ng.h/mL, *p* = 0.002). These results confirmed the unpredictable and complex pharmacokinetic interactions between SRL and CSA in kidney transplant recipients [9].

In two phase II trials in patients receiving AZA/ST [10] or MMF/ST [11] and no induction therapy, higher incidence of acute rejection was observed in those treated with SRL compared to those receiving CsA. The lack of adequate efficacy of CNI-free regimens without induction therapy, along with the complex synergistic interaction between CsA and SRL, led to studies exploring elimination or minimization of CsA, to obtain maximal early efficacy for the prophylaxis of acute rejection and prevention of long-term CsA-associated toxicities [12, 13]. Our multicenter national phase 4, open-label, randomized (1:1) trial conducted in nine centers investigated the safety and efficacy of concentration-controlled use of SRL and CsA followed by CsA minimization or elimination beginning at week 13 [14]. At 12 months, there were no differences in renal function (61.08 vs. 65.24 mL/min, *p* = 0.132), incidence of biopsy-confirmed acute rejection (14.3 vs. 22.5 %, *p* = 0.152). There were no differences in the overall rate of study-drug discontinuation (32.4 vs. 36.3 %, *p* = 0.562) but more patients discontinued because of lack of efficacy/graft loss in the CsA elimination group (4.8 vs. 14.7 %, *p* = 0.018). In summary, CsA minimization or elimination offers treatment options for de novo renal allograft recipients [15, 16]. Although few studies have systematically investigated the ideal CsA exposure in combination with SRL, it has been suggested that up to 80 % reduction can be used with even superior efficacy compared to full CsA exposure, with or without SRL [17].

The growing number of kidney transplant recipients receiving TAC and MMF prompted us to compare it with the combination of TAC and SRL [18]. Kidney transplant recipients receiving TAC-based immunosuppressive regimen were randomized to receive fixed daily doses of MMF (2 g/day) or SRL (one loading dose of 15 mg, 5 mg/day till day 7, and 2 mg/day thereafter) without induction therapy. Mean whole blood TAC concentrations among patients receiving MMF or SRL were similar at 1 (10.4 ± 4.0 vs. 9.8 ± 3.2 ng/mL) and 12 (6.9 ± 2.3 vs. 7.1 ± 2.5 ng/mL) months. Mean plasma mycophenolic acid (MPA) trough concentrations were 2.9 ± 1.7 and 3.6 ± 2.2 mg/L, and mean whole blood SRL trough concentrations were 4.2 ± 1.8 and 6.6 ± 3.5 ng/mL, at 1 and 12 months, respectively. No differences were observed in the incidence of biopsy-confirmed acute rejection (12 vs. 14 %, *p* = 1.000). Patients receiving SRL showed higher mean serum creatinine (1.6 ± 0.5 vs. 1.4 ± 0.3 mg/dL, *p* = 0.007), higher proportion of patients with proteinuria (52.0 vs. 10.7 %, *p* = 0.041), higher mean urinary protein concentrations (0.3 ± 0.5 vs. 0.1 ± 0.2 g/L, *p* = 0.012), higher mean cholesterol concentration (217 vs. 190 mg/dL, *p* = 0.030), and higher proportion of patients prematurely discontinued from randomized therapy (26 vs. 8 %, *p* = 0.031). Similar findings were observed in large international multicenter trials [19, 20]. Similar to the CsA clinical experience, more recent studies have shown that lower TAC concentrations should be used combined with SRL [21] and that TAC discontinuation in patients receiving SRL was associated with increased incidence of acute rejection [22].

During mid-2000s, with the approval of the anti-IL-2R blockers for induction therapy, several trials of ST or CNI avoidance or withdrawal were conducted, all with the purpose of improving long-term tolerability and safety of immunosuppressive regimens. With the premise from previous studies, we decided to explore whether SRL would be effective in CNI-free or ST-free regimen. We first evaluated the efficacy and safety of two CNI-free regimens in low-risk recipients of one haplotype living-related kidney transplants [23]. Immunosuppression consisted of TAC, AZA, and ST vs. two doses of daclizumab, MMF, and STvs. two doses of daclizumab, MMF, SRL, and ST. At 12 months, the incidence of BCAR was higher in patients receiving CNI-free regimes (10.5 vs. 48.5 vs. 24.0 %, *p* <0.01, respectively). In patients of black ethnicity, the incidence of acute rejection was higher in the MMF/ST group (25 vs*.* 83.3 vs*.* 20 %, *p* = 0.055), respectively. There were no differences in mean calculated creatinine clearance at 12 months (58.9 ± 14.0, 56.9 ± 16.4, 59.3 ± 21.4 mL/min). Overall incidence of post-transplant diabetes mellitus (3.3 %) and cytomegalovirus disease (4.3 %) was similar in all groups. It was then clear that CNI-free regimen, with MMF or SRL/MMF, would not sustain low rates of acute rejection, as demonstrated in multicenter international trials [24–26].

Subsequently, we explored early discontinuation of either CNI or ST. Recipients of first renal transplant received SRL, TAC, and ST without induction therapy and were randomized to undergo ST (SRL/TAC) or TAC (SRL/ST) withdrawal 3 months after transplantation [27]. Lower mean whole blood SRL trough concentration was observed in patients receiving SRL/TAC compared to SRL/ST (11.5 ± 3.0 vs. 15.3 ± 4.5 ng/mL). No differences were observed in the incidence of BCAR (4.2 vs. 9.5 %). Mean calculated creatinine clearance was comparable (60 ± 11.5 vs. 63.4 ± 10.5 mL/min), and no significant differences were observed in the proportion of patients with proteinuria at 12 months (20 vs. 30.7 %, *p* = 0.629), respectively. We observed a higher incidence of lymphocele or lymphorrhea (13 vs. 4.1 %), proteinuria (13 vs. 8.3 %), graft dysfunction (17.3 vs. 4.1 %), stomatitis (30.4 vs. 8.3 %), headache (56.5 vs. 33.3 %), leucopenia (8.6 vs. 0 %), thrombocytopenia (8.6 vs. 4.1 %), dyslipidemia (78.2 vs. 66.7 %), and CMV infection (4.3 vs. 0 %) in SRL/ST group. The incidence of NODAT (10.6 %) was similar between groups. Higher mean cholesterol concentration was observed in the SRL/ST group (191.9 ± 63.3 vs. 241.6 ± 61.5 mg/dL, *p* = 0.019). Treatment discontinuation due to adverse events occurred in 12.5 % of patients in SRL/TAC group and 21.7 % in SRL/ST group. Within 12 months of observation, our study was unable to detect any significant difference in major transplant outcomes comparing CNI and ST elimination strategies. In this context, a recent review concluded that the benefits of minimizing immunosuppression, either ST or CNI, must be weighed against the risks of precipitating acute rejection or chronic allograft dysfunction [28].

These exploratory studies conducted in de novo kidney transplant recipients allowed us to perform a 10-year retrospective analysis of pooled data from patients included in prospective randomized trials in de novo kidney transplant recipients receiving CNI combined with SRL (*n* = 329) or AZA/MMF (*n* = 124). We did not observe differences in patient (89.1 vs. 91.1, *p* = 0.766), graft (72.9 vs. 76.6 %, *p* = 0.709), and biopsy-confirmed acute rejection-free (78.1 vs. 79.0 %, *p* = 0.976) survivals, respectively. The incidence of CMV infection was lower (6 vs. 11 %, *p* = 0.024), but treatment discontinuation was higher among patients receiving SRL (66 vs. 31.5 %, *p* <0.001), respectively. At 5 years, mean estimated glomerular filtration rates were comparable (57.4 ± 18.6 vs. 57.0 ± 19.2 mL/min, *p* = 0.111) but the proportion of patients with proteinuria was higher among patients receiving SRL (44 vs. 19 %, *p* <0.001), respectively [29].

## Critical analysis

The interest for the clinical use of SRL and CNI in de novo kidney transplant recipients has reduced since its approval in early 2000. The basic reason behind this observation is perhaps the lack of a thorough understanding of the interaction between these two drugs. Although the pharmacokinetic interaction between SRL and CsA was anticipated, high doses and concentrations of both SRL and CsA or TAC were used initially, leading to a disproportionally higher incidence of adverse events, poor tolerability, and ultimately drug discontinuation. Key adverse events of this drug combination have been associated with higher concentrations of both drugs, namely, wound healing [30] and inferior renal function [31]. Not surprisingly, two registry analyses showed inferior graft survival in patients receiving SRL combined with CsA [32] or TAC [33].

Alternatively, CNI avoidance and withdrawal trials were implemented to avoid or minimize this drug interaction. A recent systematic review and meta-analysis of randomized controlled trials showed higher incidences of acute rejection but superior renal functions with no differences in patient or graft survival were observed at 1 year after transplantation [34]. Nevertheless, a registry analysis confirmed that a CNI-free immunosuppressive regimen consisted of SRL/MMF combination was associated with inferior renal transplant outcomes compared to CNI combined with SRL or MMF [35]. Two main reasons emerge from this observation. First, SRL and MMF share similar profile of adverse events such as gastrointestinal and bone marrow toxicities. Second, recent data have suggested the increased risk of acute rejection or chronic antibody-mediated rejection in presents with suboptimal CNI exposure [28]. Therefore, more studies exploring different drug concentrations are needed to define proper doses of both drugs associated with a more favorable short- and long-term efficacy/safety profile [36]. Nevertheless, prospective trials are required to define therapeutic concentrations for both drugs associated with best efficacy/toxicity ratios.

## Conversion strategies

Our first experience with conversion from CNI to SRL occurred during the Sirolimus Renal Conversion Trial (CONVERT) [37]. This study explored late conversion, mean time after transplantation of 37 months, from CNI to SRL. The primary efficacy endpoint, Nankivell GFR in the intent-to-treat (ITT) population 12 months after randomization, was comparable (59.0 and 57.7 mL/min) in the cohort of patients with baseline GFR more than 40 mL/min. Conversion was associated with no difference in the incidence of acute rejection, increased urinary protein excretion, and a lower incidence of malignancy compared with CNI continuation. Treatment discontinuation was higher in SRL conversion versus CNI continuation patients at 12 (15.7 vs. 9.5 %, respectively, *p* = 0.013) and 24 (25.8 vs. 20.0 %, *p* = 0.070) months. Superior renal function was observed among patients who remained on SRL through 12 to 24 months, particularly in the subgroup of patients with baseline GFR more than 40 mL/min and proteinuria less than or equal to 0.11.

Following the initial promising data of the Spare the Nephron trial [38], we designed our multicenter, prospective, open-label, national trial with planned conversion from TAC to SRL 3 months after kidney transplantation. Of 297 patients initially treated with TAC, MPS, and ST, 283 patients reached 3 months of whom 97 were converted to SRL, 107 were maintained on TAC, and 79 were patients receiving TAC without criteria to undergo intervention. There was no difference in the primary objective, superior estimated glomerular filtration rate in the SRL group at month 24 in the intention-to-treat population (66.2 ± 25.3 vs. 70.7 ± 25.1mL/ min, *p* = .817). There was also no difference in the severity of chronic sclerosing lesions scores in 24-month protocol biopsies. Higher mean urinary protein-to-creatinine ratio (0.36 ± 0.69 vs. 0.15 ± 0.53, *p* = 0.03) and higher incidence of treated acute rejection between months 3 and 24 (13.4 vs. 4.7 %, *p* = 047) were observed in SRL compared to TAC group [39]. Interestingly, comparable results were observed in a multicenter international trial with similar design [40].

## Critical analysis

Late conversion from CNI to SRL has not been consistently associated with significant improvement in renal function. Nevertheless, the trials identified risk factors associated with poor clinical therapeutic response, including the level of renal function, proteinuria, and structural damage [41]. Early conversion trials, while excluding patients with these risk factors, were associated with loss of efficacy, lower tolerability and increased incidence of adverse events. In one study, 192 kidney transplant recipients receiving CsA, MMF, and ST were converted to SRL (*n* = 95) at 3 months or continued CsA (*n* = 97) and underwent planned ST discontinuation at month 8 (CONCEPT) [42]. At 12 months, the incidence of acute rejection was higher (17 vs. 8 %, *p* = 0.071) but renal function was superior in patients converted to SRL (68.9 vs. 64.4 mL/min/1.73 m^2^, *p* = 0.017) compared to those maintained on CsA. Interestingly, the incidence of subclinical inflammation in protocol biopsies performed at 12 months (30.6 %) was higher in the SRL compared to CsA group (45.2 vs. 15.3 %) and associated with lower estimated glomerular filtration rate (50.8 ± 13.3 vs. 57.7 ± 16.3 mL/min/1.73 m^2^, *p* = 0.035) at 30 months [43]. Furthermore, no significant differences were observed in the incidence of interstitial fibrosis comparing both groups [44]. In an extension study, renal function was superior in SRL (*n* = 77) compared to CsA (*n* = 85) group at 48 months (62.6 vs. 57.1 mL/min/1.73 m^2^, *p* = 0.013) [45].

Taken together, when choosing this immunosuppressive strategy, one needs to balance the risks of early subclinical and clinical rejection and the long-term benefits of superior renal function. Better understanding of risk factor for acute rejection, using more robust diagnostic methods, may increase the success of these strategies. Low tolerability may be associated with factors intrinsic to the drug (aftous ulcers, dyslipidemia, peripheral edema) but others emerge as a consequence of overlapping toxicity profile of MPA and SRL [46]. Better partners such as belatacept may improve the long-term safety of SRL [47]. More studies aiming to investigate SRL-associated proteinuria using different immunosuppressive strategies after kidney transplantation is essential to increase the safety of these regimens [48]. In this direction, a multicenter prospective trial demonstrated that the use of ramipril prior to early conversion from CNI to SRL is associated with lower incidence of proteinuria during the first 12 months [49].

The association of SRL with lower incidence of viral infections is opened for various types of investigation [50]. Significant reductions in the incidence of CMV [51] and BKV [52] infections have been reported in patients receiving SRL. Cancers with higher relative incidence among kidney transplant recipients compared to the general population are associated with viral infection such as Kaposi sarcoma (herpesvirus 8), non-Hodgkin lymphomas (EBV), and skin cancer (HPV) [53]. The association between SRL and lower incidence of cancer will be explored in the future [54], as death due to cancer contributes increasingly to the overall death rate among kidney transplant recipients (http://www.anzdata.org.au/anzdata/AnzdataReport/31stReport/Ch10CancerReport.pdf). The association of SRL and reduced recurrence of non-melanoma skin cancer was well documented in three independent international multicenter trials [55–57]. Nevertheless, the benefit observed was mitigated by the low tolerability of the SRL-containing immunosuppressive regimen.

## Future perspectives

It is surprising that even after 15 years of clinical use, SRL-based immunosuppressive regimens are still evolving. More importantly, several areas of research are still open for future studies. Comprehensive understanding the complex pharmacokinetic and pharmacodynamics interaction between SRL and CNI will be decisive to investigate and define target concentrations for both classes of drug, either in de novo or as a conversion strategy. In experimental models, dose-dependent biochemical metabolite patterns differences were observed in the brain [58] and in the kidney [59] when combining SRL or EVR with CsA, suggesting that SRL potentiates CsA-induced mitochondrial dysfunction. Nevertheless, whether this metabolic difference is translated into measurable clinical differences in outcomes or renal function is not known as there is no head-to-head comparison of these two drugs using CNI minimization strategies. This will be even more important because sotrastaurin [60, 61] and tofacitinibe [62] drug development program were prematurely terminated and no new drugs are expected to be approved for clinical use soon. The effects of SRL in reducing the incidence of viral infections and malignancies may influence transplant outcomes as long as we increase safety and tolerability of SRL-containing immunosuppressive regimens. Promising strategies include the combination of SRL with once-daily TAC or belatacept. While the first once-daily TAC formulation of TAC has already been used in the clinical setting [63], a new formulation has finished phase three trials and will soon be registered [64]. The pharmacokinetics, efficacy, and safety of the combination of SRL with once a day TAC should be investigated in future studies, including methods to measure the influence of adherence on transplant outcomes. A pilot study in de novo kidney transplant recipients receiving rabbit anti-thymocyte globulin and short course of ST showed similar efficacy and 8–10 mL/min higher glomerular filtration rate at 12 months compared with TAC/MMF [47]. Furthermore, while SRL has been associated with increasing regulatory T cells [65], belatacept has been associated with lower incidence of de novo donor-specific antibodies [66]. In summary, although several SRL-contained immunosuppressive strategies have already been tested in solid organ transplantation over the last 20 years, many more studies are needed to allow our patients to fully benefit from this therapy in the future.
